# Bacteriophage Therapy Against *Klebsiella Pneumoniae*

**DOI:** 10.3390/microorganisms14010201

**Published:** 2026-01-15

**Authors:** Weijia Ding, Yicheng Wen, Liang Chen, Hong Du

**Affiliations:** 1 Department of Clinical Laboratory, the Second Affiliated Hospital of Soochow University, Suzhou 215004, China; weiijia_ding@126.com (W.D.);; 2 Department of Pharmacy Practice, School of Pharmacy and Pharmaceutical Sciences, University at Buffalo, Buffalo, NY 14214, USA

**Keywords:** *Klebsiella pneumonia*, hypervirulent, carbapenem resistant, phage, phage therapy

## Abstract

Antibiotic resistance is arguably one of the greatest threats to global health today. The worldwide emergence of multidrug-resistant and hypervirulent Klebsiella pneumoniae underscores the urgent need for alternative treatments. Bacteriophages (phages) are considered one of the most promising alternatives to address this crisis. In this review, we summarize current knowledge of phage–host interactions and highlight recent advances in phage therapy against K. pneumoniae, including phage cocktails, antibiotic combination therapy, and treatments based on phage-derived proteins. Despite their tremendous therapeutic potential, significant challenges remain. We therefore also discuss strategies to optimize phage research and recent innovations in the field.

## 1. Introduction

*Klebsiella pneumoniae* is an important Gram-negative opportunistic pathogen that primarily resides in the human gastrointestinal tract. It can cause a wide range of infections, including urinary tract infections, bacteremia, pneumonia, and liver abscesses [1]. Inappropriate or excessive use of antimicrobial drugs during treatment could contributed to the emergence of drug-resistant strains [2]. As a member of the ESKAPE pathogens—which include *Enterococcus faecium, Staphylococcus aureus, K. pneumoniae, Acinetobacter baumannii, Pseudomonas aeruginosa,* and *Enterobacter* species [3]—*K. pneumoniae* poses a serious threat to global public health.

Bacteriophages, noted for their high specificity, strong lytic activity, safety, and low cost [4], represent promising therapeutic agents against multidrug-resistant *K. pneumoniae*. Although phage therapy has shown considerable potential in both research and clinical applications, its limitations should not be overlooked [5]. To address the narrow host range resulting from phage specificity, strategies such as phage cocktails and genetic engineering can be employed. These approaches can also help delay the development of phage resistance in bacteria. In addition, phage-derived proteins have been extensively investigated for their therapeutic applications [6].

## 2. High-Risk *K. pneumoniae* Pathotypes

*K. pneumoniae* is an increasingly dangerous pathogen that poses a major threat to public health, particularly in immunocompromised individuals and patients undergoing invasive treatments. Traditionally, *K. pneumoniae* has been associated with pneumonia, intra-abdominal infections, urinary tract infections, and bloodstream infections [7]. However, in recent decades, more virulent and drug-resistant strains have emerged, greatly complicating clinical management. Two major pathotypes of *K. pneumoniae* are defined by their primary mechanism of pathogenesis: the carbapenem-resistant pathotype (CRKP), which is marked by drug resistance, whereas the hypervirulent pathotype (hvKP) is distinguished by its high invasiveness [8].

### 2.1. Carbapenem-Resistant K. pneumoniae (CRKP)

Carbapenem-resistant *K. pneumoniae* (CRKP) has become a major threat in clinical settings due to its high prevalence in healthcare-associated infections and its multidrug-resistant (MDR) phenotype. These strains are frequently linked to nosocomial outbreaks, invasive medical procedures, and infections in immunocompromised patients, leading to high morbidity and mortality rates [9]. Resistance to carbapenems, which are considered the last line of defense, makes treatment particularly challenging. Moreover, the global dissemination of carbapenemase-producing *K. pneumoniae* has further aggravated the situation. Beyond carbapenems, these strains have also acquired resistance to other critical antibiotics, including polymyxins, tigecycline, and aminoglycosides, thereby further narrowing the available therapeutic options [10].

### 2.2. Hypervirulent K. pneumoniae (hvKP)

Unlike the carbapenem-resistant pathotype (CRKP), hypervirulent *K. pneumoniae* (hvKP) exhibits significantly greater virulence and can infect both immunocompromised and otherwise healthy individuals [11]. The first case of hvKP was reported in 1986 in a Taiwanese patient who developed a liver abscess complicated by endophthalmitis [12,13]. Since then, hvKP infections have been increasingly reported worldwide. The virulence of hvKP is primarily attributed to its thick capsular polysaccharide layer and efficient iron acquisition system [14]. The hypercapsule phenotype, regulated by genes such as *rmpA*, protects the bacteria from phagocytosis [15], while the hypermucoviscosity phenotype is another hallmark [16]. Additionally, hvKP produces more siderophores than CRKP, allowing it to acquire iron more effectively from the host—an essential factor for bacterial survival and growth [17]. By virtue of these enhanced virulence mechanisms, hvKp gains the capacity to proliferate robustly, persist tenaciously in host niches, and ultimately inflict severe tissue damage [18,19].

**Figure 1:**
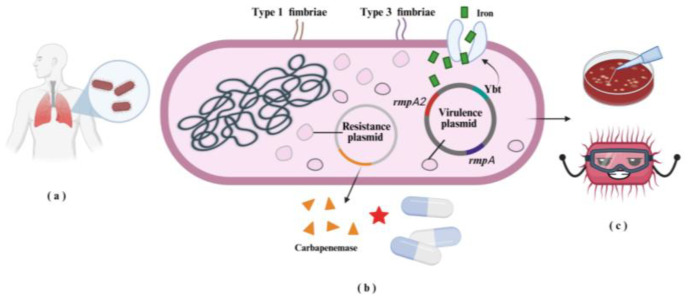
High-Risk *K. pneumoniae* Pathotypes: (**a**) *K. pneumoniae* is a primary pathogen causing pneumonia in the human respiratory system. (**b**) Carbapenem-resistant hvKP (CR-hvKP) carries both resistance and virulence plasmids. Carbapenem antibiotics are inactivated through enzymatic hydrolysis by carbapenemases. (**c**) The strain also develops a hypermucoviscous phenotype, enhancing its tolerance to external stressors.

Of particular concern is the emergence of carbapenem-resistant hvKP (CR-hvKP), which combines hypervirulence with carbapenemase-mediated resistance [20] (Figure 1). These convergent strains are especially problematic because they evade last-line antibiotics while simultaneously driving severe disease progression, particularly in healthcare settings where immunocompromised patients are at high risk [21].

Given the limited treatment options available, there is an urgent need for alternative therapeutic strategies to effectively control *K. pneumoniae* infections and relieve growing public health pressure.

## 3. Phage Therapy

Against the backdrop of increasing antimicrobial resistance and limited antibiotic options, phage therapy has gained significant attention in recent years. Research has demonstrated its potential against multidrug-resistant pathogens, including *K. pneumoniae*, with several clinical trials already reporting promising outcomes [22].

### 3.1. What Are Phages?

In 1915, bacteriologist Frederick W. Twort observed an acute infectious disease of micrococci and first described viruses that infect bacteria [23]. Two years later, French scientist Félix d’Hérelle independently discovered and isolated a Shigella-killing agent, which he named “bacteriophage.” Phages are the most abundant and widely distributed type of virus, commonly found in bacteria-rich environments such as the intestines of animals [24,25]. They can only reproduce inside living bacteria, with strict host specificity. This targeted infection allows them to multiply within specific bacterial species and ultimately kill their hosts, making them a potential tool for treating bacterial infections [26].

Structurally, phages consist of two main parts: a head and a tail. They are composed of nucleic acids (DNA or RNA) encased in protective proteins [27]. These proteins determine the phage’s morphology and surface properties. Phage genomes may be double- or single-stranded and can exist in either circular or linear forms [28].

### 3.2. Life Cycles and Their Potential Therapeutic Applications

**Figure 2:**
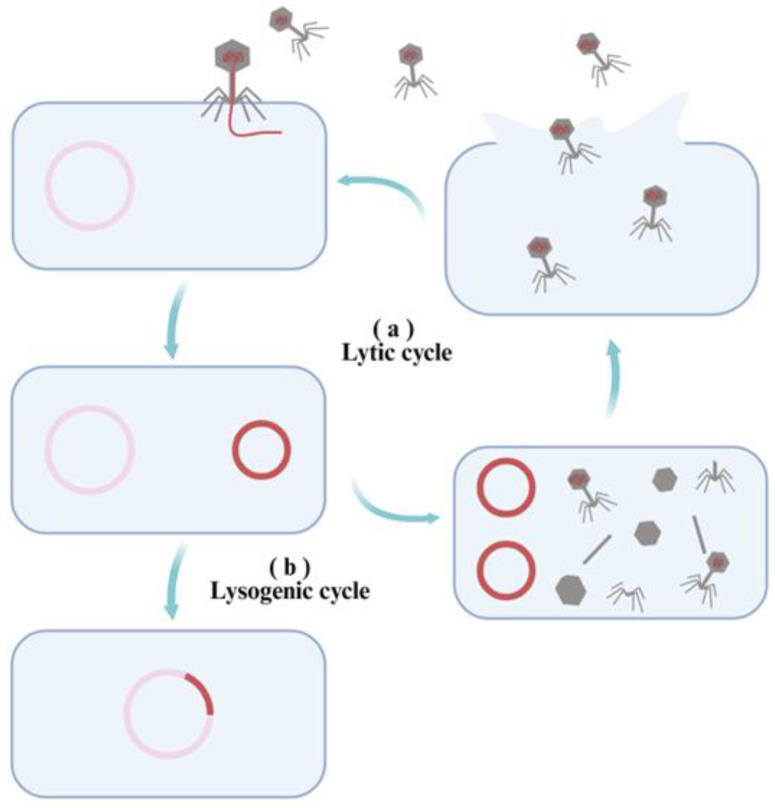
The types of phage life cycles: (**a**) The lytic cycle consists of sequential stages—adsorption, penetration, biosynthesis, maturation, and lysis. (**b**) The lysogenic cycle involves integration of the phage genome into the host chromosome, establishing a dormant state.

Phages are broadly classified into virulent and temperate types, based on their reproductive behavior [29]. Each follows a distinct life cycle (Figure 2), which directly influences their therapeutic applications.

#### 3.2.1. Virulent Phages (Lytic Cycle)

Virulent phages exclusively undergo the lytic cycle, which consists of three main stages: adsorption and penetration, biosynthesis, and lysis. In this cycle, the phage attaches to the host cell surface, injects its genetic material, and hijacks the host’s machinery to synthesize and assemble new phage particles [30]. Once enough particles have accumulated, the bacterial cell lyses, releasing progeny phages to infect new hosts.

Clinically, virulent phages are regarded as one of the most promising candidate therapies for treating infections caused by *K. pneumoniae* and other multidrug-resistant (MDR) pathogens [31]. The core advantage of virulent phages lies in their ability to rapidly and efficiently eliminate bacterial populations. By replicating inside bacterial cells and inducing lysis, they can swiftly reduce bacterial populations, a particularly valuable property for treating infections. Moreover, virulent phages often display a high degree of host specificity, enabling them to target pathogenic bacteria while sparing beneficial microbiota, thereby minimizing potential side effects compared with broad-spectrum antibiotics [32]. Therefore, phage therapy exclusively utilizes virulent phages for MDR bacterial infections.

#### 3.2.2. Temperate Phages (Lysogenic Cycle)

Temperate phages typically follow the lysogenic cycle as their primary lifestyle: their genome integrates into the host chromosome to form a prophage, and the host cell becomes a lysogen. In this state, the phage is stably inherited during bacterial division, while retaining the ability to switch to the lytic cycle in response to environmental signals [33]. Although this trait allows phages to persist long-term within bacterial populations, it also limits their direct use in clinical settings where rapid bacterial killing is required.

Embedded within this adaptive host–phage dynamic, temperate phage integration continues to reconfigure the biological profile of the host. For example, certain *E. coli* prophages encode diverse defense systems that specifically protect lysogens from infection by other temperate phages [34]. More importantly, this integration can directly enhance the fitness and pathogenicity of the pathogen. Studies have shown that in chronic lung infections, pathogens carrying specific prophages (such as *Pseudomonas aeruginosa*) often exhibit enhanced biofilm formation, antibiotic tolerance, and immune evasion capabilities [35].

Traditionally, temperate phages have been approached with caution in therapeutic contexts due to their lysogenic potential and possible carriage of virulence genes. Yet growing evidence suggests rationally designed temperate phages function as targeted delivery vehicles, with substantial potential for chronic and recurrent infection therapy [36].

#### 3.2.3. Pseudolysogeny Cycle

Phage genomes are capable of persisting in a pseudolysogenic state across diverse bacterial populations. In this state, the phage genome does not replicate synchronously with the host and is unevenly distributed among daughter cells. It is often associated with limited host metabolism or environmental stress [37].

Notably, pseudolysogeny is closely linked to the development of phage resistance in bacteria. For example, in *P. aeruginosa*, pseudolysogeny—combined with sequential mutations—can lead to stable and broad resistance against multiple virulent phages [38]. Similar mechanisms may exist in other pathogens. Certain putative pseudolysogeny-related phage genes in *Cutibacterium acnes* have been shown to confer superinfection resistance [39].

This mechanism carries profound implications for phage therapy. Pseudolysogeny may act as a latent survival strategy that endows bacteria with tolerance to phage predation. This not only delays bacterial eradication and elevates the risk of infection recurrence, but also potentially facilitates the evolution of phage-resistant phenotypes.

### 3.3. Advantages over Traditional Antibiotic Therapy

#### 3.3.1. Targeted Killing

Phages infect only specific bacterial species or even specific strains, without disrupting the body’s normal flora (e.g., intestinal microbiota) [40]. This specificity helps reduce the risk of bacterial imbalance, such as diarrhea or secondary fungal infections, commonly caused by broad-spectrum antibiotics.

#### 3.3.2. Self-Limiting

Following the elimination of susceptible bacterial populations, phage replication capacity diminishes, resulting in a natural decline in viral titers. Subsequently, phages are progressively cleared via immune-mediated mechanisms and systemic circulation [41]. In nebulized inhalation phage therapy targeting *P. aeruginosa*-associated pulmonary infections in cystic fibrosis patients, this property of phages exhibits precise compatibility with the clinical treatment paradigm. Specifically, after phages mediate a substantial reduction in target bacterial burdens in the lungs, they lose their obligate bacterial hosts and thus do not persist or accumulate in lung tissues or airway compartments [42].

#### 3.3.3. Auto-Dosing

Phages can adaptively evolve in response to environmental and host changes, enabling continued infection of bacteria [43]. Because they replicate and maintain activity as long as the target bacteria are present, the need for repeated dosing is reduced. In a phage therapy experiment targeting *P. aeruginosa* skin infection, topical application of phages significantly reduced the bacterial load in the tissue, and a single dose was sufficient to achieve a therapeutic effect [44].

#### 3.3.4. Biofilm-Busting

Phages can penetrate and disrupt bacterial biofilms (e.g., in chronic wound infections or catheter-associated infections [45,46]), whereas antibiotics are typically far less effective against biofilm-embedded bacteria. This feature has been further corroborated in research conducted on *Staphylococcus aureus*: novel polyvalent phages demonstrate the ability to target multiple genotypic strains and effectively disrupt complex biofilms in vitro via enzyme-assisted penetration and broad-spectrum lytic activity [47].

#### 3.3.5. Strain-Tailored

By isolating a patient’s infectious strain and matching it with a specific phage, phage therapy enables a form of “precision medicine,” particularly beneficial for patients with complex or chronic infections [48]. Personalized phage therapy can significantly enhance the eradication of target bacteria in such cases [49]. However, further studies are required to pave the way for broader clinical translation.

## 4. Research on Phage Therapy Against *K. pneumoniae*

**Figure 3:**
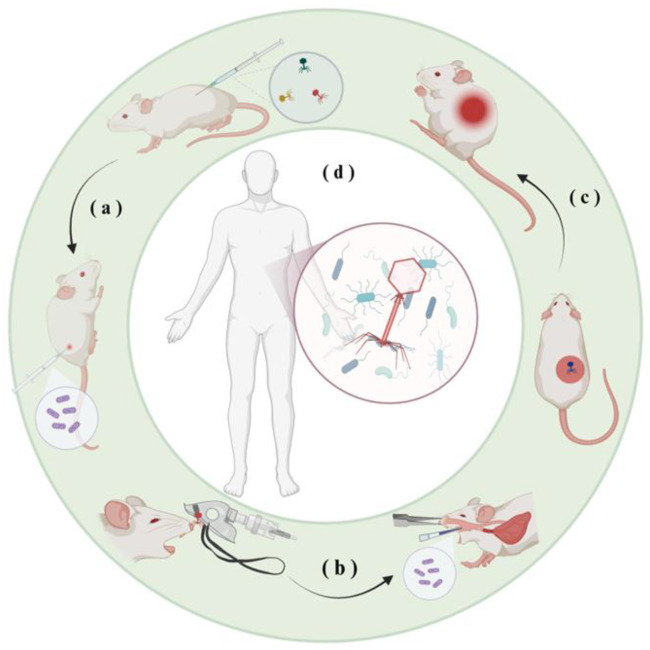
Research on phage therapy against *K. pneumoniae*: (**a**) In animal infection models, bacteriophages are administered intravenously for therapeutic intervention. (**b**) A murine pneumonia model is established, with phages delivered via nebulized inhalation. (**c**) Topical application of bacteriophages to wound sites is used to treat infected animals. (**d**) Multiple clinical cases have demonstrated that precisely matched phage therapy significantly reduces bacterial load in treatment-refractory *K. pneumoniae* infections, providing a lifesaving therapeutic alternative.

Given the rising threat of CRKP and hvKP, the need for alternative therapies has become urgent. In this context, phage therapy has emerged as a promising strategy, with recent studies and clinical trials demonstrating its efficacy against *K. pneumoniae* infections in both animal models and select clinical cases (Figure 3).

### 4.1. The Receptors and Mechanisms

The process of recognition and binding to specific bacterial surface receptors is the fundamental first step for phage-targeted lysis [36]. Different receptors fulfill distinct roles, and alterations in receptor structure are a key driver of bacterial resistance to phages [50]. Therefore, a deep understanding of these receptor-level interactions is not merely a mechanistic detail, but the very cornerstone for designing effective clinical phage therapies. It directly dictates critical practical considerations such as host range determination, cocktail formulation to preempt resistance, and the rational selection of phages for treating specific bacterial infections.

#### 4.1.1. Core Receptor Type: Specific Recognition by Capsular Polysaccharide (CPS)

The capsular polysaccharide (CPS) is a key virulence factor of *K. pneumoniae*, located on the outermost layer of the bacterium. It enhances pathogenicity by resisting phagocytosis and promoting biofilm formation [51]. Traditional serotyping has identified at least 80 capsular serotypes, with significant differences in structure and genetic synthesis mechanisms. HvKP are primarily associated with the K1 and K2 serotypes [52], while CRKP are mainly linked to the Asian ST11 clone (KL64) and the Euro-American ST258 clone (KL107), which have become important markers of nosocomial resistance [53]. Notably, the synthesis of all serotypes is governed by a single pathway (the Wzx/Wzy-dependent pathway), with all relevant genes located within the CPS locus [54]. Correspondingly, phage recognition and infection of specific serotypes depend on domains of their tail fibers or spike proteins, which achieve highly specific targeting by binding to unique epitopes on the CPS surface [55].

As an example, a novel lytic phage, P01 (genus Taipeivirus, family Ackermannviridae), demonstrates a narrow host range, lysing only ST11-KL64 CRKP. Notably, it encodes two tail spike proteins and a unique tail fiber protein. This distinctive complement of tail proteins is likely the molecular mechanism underlying its specific CPS recognition and targeted infectivity [56]. The lytic phage TUN1 (Autographiviridae), isolated from an ICU wastewater sample, specifically targets and degrades the K64 capsule of *K. pneumoniae*. This activity is likely mediated by a depolymerase encoded by the gene gp47 [57].

#### 4.1.2. Alternative Targets: Synergistic Mediation by LPS and Outer Membrane Proteins

Besides CPS, lipopolysaccharide (LPS) and outer membrane proteins (OMPs) also serve as major receptors for phage adsorption in *K. pneumoniae* [58]. As a key component of the outer membrane in Gram-negative bacteria, the outermost O-antigen chain of LPS is often specifically recognized by phages. Outer membrane proteins, which are β-barrel proteins embedded in the bacterial outer membrane, not only function as channels for substance transport but also act as critical portals for phage invasion [59].

For instance, the phage φKO1-1 targets the O1 lipopolysaccharide of Klebsiella pneumoniae. When used in combination with φK64-1, a phage that targets the capsular polysaccharide, this cocktail partially decolonized mice intestines carrying CRKP [60]. The phage GH-K3 mediates its efficient infection of K7 Klebsiella pneumoniae by specifically binding to the OmpC protein on the outer membrane [61]. Phage ФNJS1 utilizes FepA as its primary, irreversible receptor and the LPS O-antigen as a secondary, reversible binding site. However, elongated O-antigen chains can spatially hinder phage access to these receptors, thereby inhibiting infection [62].

#### 4.1.3. The Path from Receptor Variation to Phage Resistance

*K. pneumoniae* evades phage infection through structural and expressional alterations of phage receptors such as CPS [63], mediated by genetic or epigenetic changes. Two principal CPS-related resistance mechanisms include: (1) loss of capsule synthesis due to mutations in genes like wza, wzb, and wzc, which eliminates phage binding sites; and (2) chemical modifications of CPS sugar chains (e.g., methylation or acetylation) that mask phage recognition by altering spatial and electrostatic properties of the capsule [64,65,66]. Similarly, *K. pneumoniae* develops phage resistance through synergistic alterations of multiple receptor systems. For instance, while phage hvKpP3 utilizes CPS as its primary attachment receptor and LPS does not directly mediate adsorption, deletion of the wcaJ gene disrupts LPS synthesis and significantly impairs phage adsorption efficiency. Further studies revealed prevalent loss-of-function mutations in glycosyltransferase genes among phage-resistant strains, indicating that defective LPS structures may confer host resistance by interfering with phage recognition of critical DNA ejection sites [67].

During clinical treatment, the aforementioned resistance mutations not only directly lead to phage inactivation but may also select for subpopulations that integrate multidrug resistance, high virulence, and strong environmental adaptability. Such evolutionary traits may confer significant intrahospital transmission advantages to variant strains, thereby exacerbating the risk of infection outbreaks and complicating control measures. This further suggests that researchers should establish a phage combination library targeting different critical receptors against the same bacterial strain. This approach represents a necessary strategy to avoid reliance on a single pathway and mitigate the risk of resistance development [19].

In response, phages can evolve structural adaptations to counteract such resistance. An adaptive mutation in the alternative tail fiber protein (ORF59) of phage ZX1Δint allows it to bind to a different epitope on the LPS O-antigen, demonstrating this evolutionary strategy [65]. This suggests that researchers can proactively address potential drug resistance in clinical treatment by constructing a phage resource library capable of recognizing common receptor variant patterns through phage-directed evolution and synthetic biology modification.

### 4.2. Explorations in Therapeutic Practice

#### 4.2.1. Efficacy in Animal Models

Increasingly, animal studies have reported significant reductions in bacterial loads and improved survival rates, providing strong evidence for the effectiveness of phage therapy against *K. pneumoniae*.

The first intranasal phage administration regimen applied in the BALB/c mouse model demonstrated that initiating treatment even 48 h post-infection still achieved significant antibacterial efficacy (5-log decrease in bacterial burden compared to the control group). Histopathological analysis of lung tissue further revealed a clear therapeutic effect: mice in the control group exhibited extensive and severe pulmonary lesions, including significant inflammatory cell infiltration, alveolar structural damage, focal hemorrhage and edema, with large amounts of bacterial colonies and inflammatory exudate visible in the bronchial lumen. In contrast, the phage-treated group showed markedly reduced severity of lung lesions, with pathological changes primarily mild to moderate and inflammatory responses effectively controlled [68].

Mice infected with ST258 were treated with phage P1 alone, P2 alone, or a combination (P1+P2). Under the optimal combination therapy (P1 + P2), the results from the mouse model infected with MDR-KP ST258 showed that the 7-day survival rate exceeded 90%, bacterial clearance in target tissues reached over 90%, and no significant treatment-related toxic reactions were observed throughout the monitoring period. This indicates that the regimen combines potent antibacterial activity with favorable safety. The combination therapy yielded the lowest incidence of phage-resistant bacteria isolated from blood and tissues. Furthermore, treatment timing was found to be more critical than dosage for therapeutic outcome: intervention at 1 h post-infection was most effective, whereas delayed treatment beyond 8 h resulted in significantly reduced efficacy [69]. In a murine model of Klebsiella pneumoniae mastitis, the phage cocktail therapy also demonstrated significantly superior efficacy compared to monophage treatments. The three isolated and combined phages (mixed in equal doses) significantly suppressed the expression of key pro-inflammatory factors, including IL-1β, TNF-α, IL-6, and PG protein, within 24 h. This demonstrates that the phage combination effectively alleviates the inflammatory response induced by *K. pneumoniae* in mouse mammary tissue [70].

More complex pathophysiological responses and long-term safety have been evaluated in rat and rabbit models. Subcutaneous injection of purified bacteriophages targeting five common clinical pathogens, including *K. pneumoniae*, in rabbits showed that short-term phage applications can effectively control acute infections, as monitored by anti-phage neutralizing antibodies [71]. In a rat respiratory infection model, NK20 treatment significantly reduced PDR-KP load in lung tissue by 4.2 log_10_ CFU/g compared to the saline control. Inflammatory cytokine levels decreased by 60%–70%, and pathological lung injury scores were lowered by 58% [17]. In a localized wound infection model, topical application of a phage preparation (10^9^ PFU/mL) to MDR-KP-infected wounds resulted in a 3.8 log_10_ CFU/cm² reduction in bacterial load by day 7, with a wound healing rate of 85.6% and no observed skin irritation [72]. These studies primarily focus on acute-phase responses, lacking systematic tracking of long-term immune activation, potential autoimmune reactions, or the in vivo pharmacokinetics of phage persistence and clearance. These gaps are crucial for evaluating its feasibility as a therapeutic strategy for chronic or recurrent infections.

While phage therapy shows remarkable potential in animal models, further optimization is required, including improvements in delivery strategies, overcoming phage resistance, and minimizing immune interference. Continued exploration and refinement of animal models will provide critical support for future clinical trials.

#### 4.2.2. Clinical Case Reports

##### Case 1 Wound Infection

Application of phage therapy in polymicrobial bone infection (2019) [73] and limb-threatening prosthetic knee *K. pneumoniae* infection (2021) [74].

A 42-year-old man admitted to the trauma unit of Hadassah-Hebrew University Medical Center with bacterial osteomyelitis was co-infected with extensively drug-resistant (XDR) *Acinetobacter baumannii* (Ab) and multidrug-resistant (MDR) *K. pneumoniae*. Despite prolonged antibiotic treatment and surgical management, the infection remained unresolved. The patient ultimately received a combined regimen of phages ΦAbKT21phi3 and ΦKpKT21phi1, which target Ab and Kp strains, along with intravenous meropenem (2 g three times daily) and colistin (4.5 million units twice daily). The efficacy of this combination had been confirmed in vitro prior to treatment. The patient's wound began to heal progressively within days of initiating phage therapy. Throughout the 8-month follow-up period, no Ab or Kp positive cultures were obtained from any sampled site.

In another case, multiple surgeries and extended antibiotic therapy failed to control persistent MDR *K. pneumoniae* infections in a 62-year-old male with diabetes mellitus and bilateral knee osteoarthritis. The patient received treatment at the Mayo Clinic CRTU, with daily intravenous infusions of 6.3 × 10^10^ phages in 50 mL of normal saline administered over 30 min on weekdays through a newly placed peripheral IV line, for a total of 40 doses. Throughout the phage therapy, the patient maintained oral minocycline (100 mg twice daily). Approximately two weeks into treatment, the patient reported mild, intermittent itching in the right lower limb, though no significant infusion-related adverse events were observed. The discomfort gradually subsided and resolved completely by the end of therapy. After treatment completion, the patient was followed for 34 weeks without complications, showing significant symptom resolution.

##### Case 2 Chronic Infection

Phage therapy in recurrent urinary tract infections (rUTI) with multidrug-resistant ESBL-producing *K. pneumoniae* (2023) [75].

A kidney transplant recipient with recurrent urinary tract infections (UTIs) received phage cocktail therapy. Following sequential liver and kidney transplantation, the patient developed complications, including transplant-associated pyelonephritis. The infection was caused by an extended-spectrum β-lactamase (ESBL)-producing *K. pneumoniae* strain, which exhibited varying degrees of resistance to multiple antibiotics, such as ciprofloxacin and fosfomycin. In September 2021, the patient began a four-week course of intravenous triple-phage cocktail therapy administered twice daily. In the six months prior to treatment, the patient had experienced four symptomatic UTIs caused by *K. pneumoniae*. During the six- to twelve-month follow-up period after treatment, the ESBL-producing strain was completely eradicated with no recurrence of infection. Following phage therapy, the bacterial isolate regained susceptibility to oral antibiotics, and the patient no longer required intravenous antibiotic treatment thereafter. This case successfully demonstrates that intravenous phage therapy can serve as a standalone treatment for drug-resistant recurrent UTIs, offering a novel therapeutic pathway for complex infections refractory to conventional antibiotics.

##### Case 3 Pulmonary Infection

Personalized phage therapy for pulmonary infection caused by multidrug-resistant (MDR) *K. pneumoniae* (2023) [76].

A patient with a multidrug-resistant *K. pneumoniae* pulmonary infection at Shanghai Public Health Clinical Center received sequential nebulized therapy with a single phage (ΦKp_GWPB35) followed by a phage cocktail (ΦKp_GWPB35 + ΦKp_GWPA139), both combined with antibiotics. Following treatment, the patient showed improvement in clinical indicators and a reduction in pleural effusion. Although sputum cultures still detected the bacteria, the strain developed significant phage resistance accompanied by a marked decrease in virulence. This case demonstrates that even without complete bacterial eradication, phage therapy can benefit patients by driving the evolution of low-virulence strains, thereby expanding the clinical value of phage-based treatment.

##### Case 4 Novel Phage Therapy Application

Use of pre-adapted phages based on phage–host interplay (2022) [77].

A patient at Erasmus Hospital presented with a chronic fracture-related infection involving a polymicrobial consortium, including pan-drug-resistant (PDR) *K. pneumoniae.* The research team employed a pre-adaptive strategy by serially passaging a lytic phage against the patient’s bacterial isolates, resulting in an optimized phage preparation with an expanded host range and enhanced lytic activity. This tailored phage was administered locally in combination with antibiotics (e.g., meropenem), achieving synergistic clearance of multiple pathogens. The combined regimen successfully controlled the infection, promoted healing of the chronic wound, and demonstrated no recurrence during follow-up. This case illustrates that pre-adapted phage therapy combined with local delivery and antibiotic synergy represents a promising strategy for managing complex biofilm infections involving PDR pathogens.

## 5. Challenges to Phage Therapy

### 5.1. Biological and Clinical Hurdles

#### 5.1.1. Narrow Spectrum

The high specificity of phages acts as a double-edged sword, offering the benefit of targeted antibacterial action while introducing two core constraints in clinical practice [78]. The first constraint is the critical reliance on diagnostic accuracy. Effective phage therapy is contingent upon the precise identification of the bacterial strain. Furthermore, many clinical infections are polymicrobial. Given the inability of narrow-spectrum phages to target multiple bacterial species simultaneously, a specific phage must be matched to each pathogen [79].

#### 5.1.2. Phage Resistance

One of the greatest challenges in phage research is the evolutionary arms race between phages and their bacterial hosts [80]. They coexist and continually coevolve in confrontation. Anti-phage mechanisms operate at various stages of the phage life cycle, including blocking adsorption, preventing DNA injection, and inhibiting replication [81] as well as utilizing bacterial population effect [82]. The host can utilize the CRISPR-Cas system and the R-M system to recognize and degrade foreign DNA or RNA. Abortive infection (Abi) is another protective mechanism, whereby bacteria sacrifice infected cells to preserve the overall population and resist phage invasion [83].

#### 5.1.3. The Immune Response of the Host

A moderate immune response may support pathogen clearance, while an excessive response can diminish efficacy or cause side effects [84]. The main immunogenic components of phages are their protein capsids, with possible contributions from nucleic acids (e.g., incompletely degraded DNA or RNA) [85]. Neutralizing antibodies can block phage binding to bacteria, reducing lysis efficiency. Furthermore, antibody- or complement-mediated clearance shortens phage half-life, necessitating higher doses or more frequent administration [86].

### 5.2. Beyond Biology Uncertainty in Application

Uncertainties remain regarding the optimal dose, routes of administration, frequency, and duration of phage therapy for both prevention and treatment of infections [87,88]. More importantly, robust systems for safe production and regulatory supervision of phages must be properly established. The transition from lab-scale phage preparation to stable, cost-effective, and mass production remains a primary bottleneck. Critical hurdles that must be overcome for commercialization include ensuring high-titer yields, eliminating impurities such as endotoxins, and maintaining long-term storage stability. There is also still room for expansion in regulatory and ethical considerations, especially in the context of global clinical deployment. From a regulatory perspective, the classification of phage therapy as a pharmaceutical product and the approval standards vary across different countries and regions, and there is a lack of a cross-regional collaborative regulatory framework, which greatly hinders its standardized promotion on a global scale [89]. At present, clinical use of phage therapy has largely been limited to compassionate use cases in critically or chronically ill patients who have exhausted all authorized therapeutic options [90]. Establishing a specialized legal framework for phage therapy may be essential to facilitate its safe and systematic integration into clinical practice. Additional concerns include patient rights to informed consent and fairness in access to personalized treatments [91]. Moreover, some patients may be deterred by the perception that phages, as viruses, are inherently unsafe. With comprehensive oversight and regulation, however, phage therapy holds promise as a valuable addition to anti-infective treatment in a safe and compliant manner.

## 6. Optimization Strategy

### 6.1. Combination Therapy

Traditionally, single-phage therapy has been applied for treatment, and many new phages are being identified for use against *K. pneumoniae* infections, such as VTCCBPA43 [68], vB_KshKPC-M [92], kpssk3 [93], vB_kpnM_17-11 [94], vB_Kpn_ZCKp20p [95], IME184 LASTA [56], SJM3 [96], vB_KpnS_SXFY507 [97], vB_KpnS_Uniso31 [98], and VB_KPM_KP1LMA [99].

One study found that the KP1LMA phage has the potential to control *K. pneumoniae* and *E. coli* ATCC 13706 strains (predominant in UTIs). However, it exhibited a very narrow host range [99]. Thus, single-phage therapy often cannot overcome this limitation. Alarmingly, using a phage alone increases the risk of resistance development. After prolonged exposure, resistant mutants can emerge due to genetic mutations impairing phage adsorption. In one clinical case, bacterial resistance did not occur during patient treatment, but was observed in *in vitro* experiments [73]. Combination therapy, therefore, offers an effective strategy to broaden host range and minimize resistance [100].

#### 6.1.1. Cocktail Treatment

A recent study isolated three phages—KPAФ1, KP149Ф1, and KP149Ф2—active against MDR *Klebsiella* isolates. These phages showed strong lytic activity and stability, and their cocktail exhibited much stronger cleavage ability compared to the single phage [101]. Another study demonstrated that cocktail phages improved the survival of *Galleria mellonella* larvae infected with *K. pneumoniae* without showing toxicity. Among three administration methods tested, prophylaxis was the most effective in killing *K. pneumoniae* in the invertebrate model [102].

Cocktail therapy not only enhances lytic efficacy but also significantly reduces the emergence of phage-resistant clones. For instance, in multidrug-resistant *K. pneumoniae* isolated from diabetic foot ulcers, rapid bacterial regrowth was observed after single-phage treatment, whereas cocktail therapy delayed the development of phage resistance and demonstrated more sustained bactericidal effects [103]. This characteristic enables it to form a more robust synergistic anti-infection strategy when combined with antibiotics.

In another approach, Chen isolated an effective phage using hypervirulent *K. pneumoniae* as a host, then collected phage-resistant variants generated during the phage–host interaction. These resistant strains were used to isolate additional phages from hospital sewage [104]. The resulting cocktail demonstrated a broad host range and prolonged bacterial killing capacity, offering new ideas for therapy.

However, cocktail therapy is not always superior. A study isolating three phages against an ST29 K54 CR-hvKP strain found no significant difference between cocktail and single-phage treatment, possibly due to a lack of synergistic effects [105]. Therefore, the effectiveness of cocktails depends not merely on the individual lytic properties of phages but on their mutual synergy [106]. Careful selection of phage combinations is thus crucial for designing effective cocktails.

#### 6.1.2. Combination Therapy with Antibiotics

Combined phage–antibiotic treatment is generally well regarded, as it can improve pathogen clearance and substantially reduce the development of phage or antibiotic resistance [107]. With increasing research, the concept of Phage-Antibiotic Synergy (PAS) has been introduced to describe the phenomenon whereby sub-lethal concentrations of certain antibiotics enhance phage replication and thereby increase bacterial killing [108].

In one study, although a three-phage mixture was sufficient to inhibit biofilm formation by *K. pneumoniae*, combining phages with antibiotics achieved complete eradication of both biofilms and bacteria [109]. Another study engineered efficient phage–antibiotic combinations and assessed their efficacy both *in vitro* and in a murine model. PAS treatments significantly enhanced the bactericidal effects of colistin and tigecycline against MDR *K. pneumoniae* strains [110].

Phage LAPAZ, a promising candidate against MDR *K. pneumoniae* with diverse capsular types, belongs to the newly formed family *Drexlerviridae* and has shown remarkable stability. Importantly, it synergized well with meropenem. When combined with lower concentrations of ciprofloxacin or meropenem, LAPAZ achieved stronger inhibition of host bacteria than either phage or antibiotic alone. Notably, synergistic phage–meropenem interactions led to complete eradication of *K. pneumoniae* infections [111]. The high efficacy of this combination has also been validated across multiple experiments (see Table 1).

A recent study further evaluated phage–antibiotic interactions against *K. pneumoniae* using diverse statistical methods (t-test, Bliss model, two-way ANOVA, and checkerboard assay). Most phage–antibiotic combinations showed synergy, while only a few antagonistic interactions were observed [112]. This highlights the need for standardized approaches to assess interactions, which would improve comparability across studies and facilitate the rational design of effective therapeutic combinations. Moreover, further research into the mechanisms underlying synergy and antagonism is required to optimize phage–antibiotic therapies.

**Table 1:** The application of combination therapy with antibiotics.

Antibiotic	Target	Phage	Results
Meropenem	Four distinct capsular types of *K. pneumoniae* (K2, K30, K38, and K58)	A newly isolated phage (vB_KpnD-LAPAZ)	• A complete bacterial eradication • Nearly half of the most resistant mutant strains increased sensitivity towards ciprofloxacin due to affecting two transport proteins [111]
Ceftriaxone	MDR-*K. p*	A new phage (vB1086)	• Strong bacteriostatic ability • Reduction in the dosage of antimicrobials • Decrease in the generation of bacterial resistance [113]
Gentamicin	K47 Serotype *K. p*	A newly isolated Phage vB_KpnM_P-KP2 belonging to “KP15 virus” family	• Bacterial eradication • Inflammation reduction in a mouse model [114]
Amikacin	XDR *K. p* having carbapenemase and colistin resistance	A phage cocktail-amikacin combination	• Remarkable reduction in bacterial burden in internal organs • Strong prophylactic efficacy in murine bacteremia in vivo [115]
Ciprofloxacin	Biofilm of *K. p*	Depolymerase-producing lytic phage (KPO1K2)	• Close support for antibiotic efficacy on the reduction in bacterial biofilm [116]

### 6.2. Phage-Derived Protein Depolymerase

Phage-derived proteins have been identified as significant reservoirs of antimicrobial agents [117]. They hold potential as effective alternatives to traditional medicines and may serve as adjunctive or standalone antimicrobials in combination with standard antibiotic regimens [118]. In recent years, phage-derived proteins—particularly depolymerization enzymes characterized by their specificity and high efficiency—have been extensively studied [119].

Depolymerases, polysaccharide hydrolases encoded by phages, specifically bind to CPS, exopolysaccharides (EPS), or LPS of host bacteria [120]. The CPS of *K. pneumoniae* is closely associated with host defense, virulence, and drug resistance [121]. By degrading extracellular polymeric substances, depolymerases facilitate phage adsorption and DNA injection [122]. Moreover, these enzymes can be engineered independently to enhance their antibacterial activity [123].

In recent years, research has increasingly targeted depolymerases of phage origin that are active against different serotypes of *Klebsiella pneumoniae* Table 2. Two studies demonstrated that ΦSRD2021 [124] and P560 [125] may serve as potential antibacterial agents against serotype K47 *K. pneumoniae* infections. Both encode depolymerization enzymes specific to capsule locus type KL47, with P560 additionally showing significant inhibitory effects on biofilm formation in mice. The capsular types K1, K2, and K57 are among the most virulent representatives of *K. pneumoniae*. Depolymerase Depo16, capable of efficiently removing the capsular polysaccharide layer, has been identified as a promising antibacterial agent against serotype K1 infection [126]. Similarly, two phages, KpV74 and KpV763 [7] found to lyse K2 *K. pneumoniae*. Their encoded depolymerase, Dep_kpv74, functions as a specific glucosidase that cleaves K2-type CPS via a hydrolytic mechanism [127]. In addition, the depolymerase gp531 from the jumbo phage RaK2 has been shown to cleave K54 CPS [128].

Despite their potential, depolymerases face challenges due to their high serotype specificity and large molecular mass, which can limit tissue penetration. One possible strategy to overcome these limitations is the design of mini-depolymerases or chimeric enzymes with broader activity spectra [88].

**Table 2:** The application of phage-derived protein depolymerase.

Target	The Serotype of *K. pneumoniae*	Depolymerase
CPS	K1	Depolymerase Depo16 encoded by vB_KpnP_ZK1 [126], KpV41, KpV475 and KpV71 [7]
	K2	Dep_kpv74 encoded KpV74 [127]
	K54	Dep_kpv74 encoded by RaK2 [128]
	K57	Depolymerase encoded by kpV767 [7]
	K47	Depolymerase encoded byΦSRD2021 [124] and P560 [125]

### 6.3. More Therapeutic Strategies (Figure 4)

**Figure 4:**
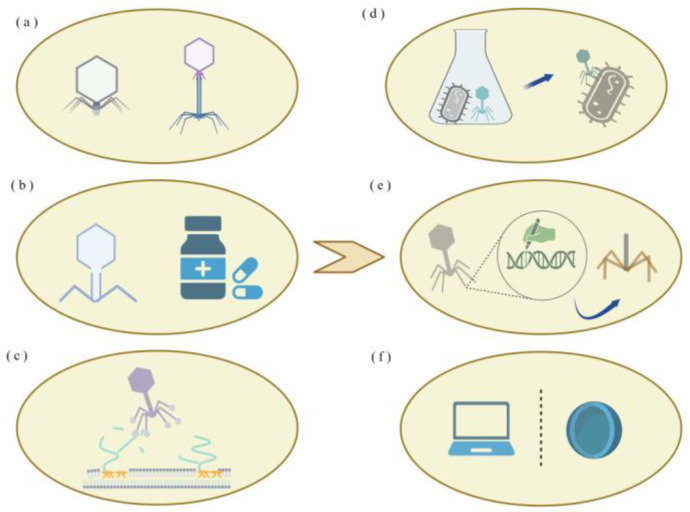
Optimization strategies for phage therapy: (**a**) Phage cocktail therapy, comprising diverse phages, is used to broaden host range specificity. (**b**) Co-administration of phages with antibiotics can increase bacterial sensitivity to the drugs, while sub-inhibitory antibiotic concentrations may enhance phage infectivity. (**c**) Depolymerases, encoded as structural proteins such as tail fibers, act on the LPS of host bacteria by cleaving polymeric compounds to facilitate phage infection. (**d**) Phages coevolve with host bacteria, generating mutants that counteract resistant strains. (**e**) Genetic reprogramming of phages enhances functional capabilities, particularly by broadening host range. (**f**) Integration of data science and nanotechnology enhances phage therapy efficacy through predictive host–interaction modeling and precision delivery systems.

To address the challenge of bacterial resistance, phage therapy is being advanced through synergistic approaches that include evolving phages, engineering them for enhanced function, and integrating materials science and computational design for optimal deployment Figure 4.

#### 6.3.1. Phage Training

##### Directed Evolution

Trained phages can be used to counteract the development of phage resistance [88]. Co-cultivating host bacteria with phages under evolutionary pressure generates mutations that allow phages to overcome bacterial defenses. This process can expand the host range, enabling infection of additional bacterial strains or species, and enhancing lytic capacity. Favor et al. [129] recently developed an accelerated evolution platform—chemically accelerated viral evolution (CAVE)—which improves phage characterization and yields variants with greater antibacterial potency.

##### Genetic Engineering

Phages can also be optimized through biotechnology approaches such as genetic engineering. Engineered phages are safer and more efficient, reduce the likelihood of resistance emergence, and may even provide personalized treatment options [130]. For example, a highly specific recombinant reporter phage, rTUN1:: nLuc, was recently developed to enable rapid detection of *K. pneumoniae* K64 cells in clinical samples such as blood and urine, facilitating identification of suitable antibiotic treatments in less than 3 h [131]. Although several engineered phages have been developed for *Escherichia coli* infection treatment, further efforts are needed to create effective engineered phages targeting *K. pneumoniae*. Pending resolution of key unknowns (genetic stability, lytic efficiency, and clinical translation), engineered phages are deployed as a targeted complement to natural phages for tackling resistant strains with scarce natural options.

#### 6.3.2. Cross-Domain Cooperation

##### Synthetic Biology

Phage efficacy may decrease under acidic conditions, such as during oral administration. Encapsulation in nanomaterials can protect phages from acid degradation, improving stability and therapeutic effectiveness [132]. Additionally, nanomaterial–phage conjugates have demonstrated synergistic antibacterial effects; one study showed promising results in reducing antibiotic resistance in wastewater [133].

##### Computer Calculations

Computational methods, including artificial intelligence, can predict key aspects of phage–host interactions, thereby guiding phage modification and cocktail design [134]. For example, Pan et al. developed a microbial heterogeneous interaction network (MHIN)-based model called PTBGRP to predict new phage–bacterial host associations and to characterize phage–phage interactions [135].

## 7. Conclusions

Phage therapy is a promising alternative to combat *K. pneumoniae*, which increasingly exhibits extensive drug resistance. Several studies have reported significant in vitro results, but more robust clinical data are needed to support its use in treating severe infections caused by CRKP. Treatment modalities for bacterial infections are advancing rapidly, encompassing monophage therapy, phage cocktails, and synergistic combination therapies. Against this backdrop, tailoring personalized treatment strategies to the phage susceptibility profile of the infecting strain, disease severity, and the patient’s comorbid health conditions has become an indispensable requirement for clinical practice.

Notably, the development of bacterial resistance to phages often entails adaptive trade-offs. For instance, when bacteria evade phage recognition through mutations in genes, LPS synthesis is regulated and surface receptor structures are altered. It is frequently accompanied by the downregulation of virulence factor expression or impaired biofilm formation. This characteristic suggests that, in specific clinical scenarios, the emergence of phage-resistant strains may simultaneously reduce the pathogenic potential of bacteria. Thereby, it offers an additional advantage for the clinical application of phage therapy.

Phage therapy is now at a critical stage of translation from laboratory research to clinical application. Challenges such as host specificity, resistance development, and large-scale production remain to be addressed. Nevertheless, its potential across precision medicine, agriculture, environmental science, and other fields is vast. With the continued integration of gene editing, artificial intelligence, and synthetic biology, phage therapy is expected to become an important pillar in the post-antibiotic era.

## Data Availability

No new data were created or analyzed in this study. Data sharing is not applicable to this article.
